# Biofilm Structure Promotes Coexistence of Phage-Resistant and Phage-Susceptible Bacteria

**DOI:** 10.1128/mSystems.00877-19

**Published:** 2020-06-23

**Authors:** Emilia L. Simmons, Matthew C. Bond, Britt Koskella, Knut Drescher, Vanni Bucci, Carey D. Nadell

**Affiliations:** a Department of Biological Sciences, Dartmouth, Hanover, New Hampshire, USA; b Department of Integrative Biology, University of California, Berkeley, Berkeley, California, USA; c Max Planck Institute for Terrestrial Microbiology, Marburg, Germany; d Department of Physics, Philipps-Universität Marburg, Marburg, Germany; e Department of Microbiology and Physiological Systems, University of Massachusetts Medical School, Worcester, Massachusetts, USA; Vanderbilt University

**Keywords:** bacteriophages, biofilm, computational biology, confocal microscopy, ecology, microfluidics, phage therapy, population dynamics, resistance evolution, spatial simulation

## Abstract

Encounters among bacteria and their viral predators (bacteriophages) are among the most common ecological interactions on Earth. These encounters are likely to occur with regularity inside surface-bound communities that microbes most often occupy in natural environments. Such communities, termed biofilms, are spatially constrained: interactions become limited to near neighbors, diffusion of solutes and particulates can be reduced, and there is pronounced heterogeneity in nutrient access and physiological state. It is appreciated from prior theoretical work that phage-bacteria interactions are fundamentally different in spatially structured contexts, as opposed to well-mixed liquid culture. Spatially structured communities are predicted to promote the protection of susceptible host cells from phage exposure, and thus weaken selection for phage resistance. The details and generality of this prediction in realistic biofilm environments, however, are not known. Here, we explore phage-host interactions using experiments and simulations that are tuned to represent the essential elements of biofilm communities. Our simulations show that in biofilms, phage-resistant cells—as their relative abundance increases—can protect clusters of susceptible cells from phage exposure, promoting the coexistence of susceptible and phage-resistant bacteria under a large array of conditions. We characterize the population dynamics underlying this coexistence, and we show that coexistence is recapitulated in an experimental model of biofilm growth measured with confocal microscopy. Our results provide a clear view into the dynamics of phage resistance in biofilms with single-cell resolution of the underlying cell-virion interactions, linking the predictions of canonical theory to realistic models and *in vitro* experiments of biofilm growth.

**IMPORTANCE** In the natural environment, bacteria most often live in communities bound to one another by secreted adhesives. These communities, or biofilms, play a central role in biogeochemical cycling, microbiome functioning, wastewater treatment, and disease. Wherever there are bacteria, there are also viruses that attack them, called phages. Interactions between bacteria and phages are likely to occur ubiquitously in biofilms. We show here, using simulations and experiments, that biofilms will in most conditions allow phage-susceptible bacteria to be protected from phage exposure, if they are growing alongside other cells that are phage resistant. This result has implications for the fundamental ecology of phage-bacteria interactions, as well as the development of phage-based antimicrobial therapeutics.

## Abstract (precis)

In the natural environment, bacteria most often live in communities bound to one another by secreted adhesives. These communities, or biofilms, play a central role in biogeochemical cycling, microbiome functioning, wastewater treatment, and disease. Wherever there are bacteria, there are also viruses that attack them, called phages. Interactions between bacteria and phages are likely to occur ubiquitously in biofilms. We show here, using simulations and experiments, that biofilms will in most conditions allow phage-susceptible bacteria to be protected from phage exposure, if they are growing alongside other cells that are phage resistant. This result has implications for the fundamental ecology of phage-bacteria interactions, as well as the development of phage-based antimicrobial therapeutics.

## INTRODUCTION

Because of the sheer number of bacteria and phages in nature, interactions between them are common (1–9). The imperative of evading phages on the part of their bacterial hosts—and of accessing hosts on the part of phages—has driven the evolution of sophisticated defensive and offensive strategies by both (10, 11). Phage resistance can evolve rapidly in well-mixed liquid cultures of bacteria under phage attack (2, 12, 13); for spatially structured environments, on the other hand, recent work has suggested that selection for phage resistance can take on very different forms due to protection of phage-susceptible cells in confined refugia (14–17). The generality of this prediction in realistic biofilm conditions is currently unknown; to address this knowledge gap, we leverage a custom biofilm-specific simulation framework and test our predictions with a microfluidics-based experimental system.

Biofilms are characteristically heterogeneous, including steep gradients in nutrient availability, waste product accumulation, oxygenation, and pH, among other factors (18, 19). Furthermore, biofilm structure can impede the movement of solutes and particles that ordinarily would pose grave threats in well-mixed liquid conditions. The extracellular matrix of Pseudomonas aeruginosa, for instance, can block the diffusion of antibiotics such as tobramycin (20, 21). Biofilm matrix secreted by Escherichia coli and P. aeruginosa can also alter phage movement (22, 23), and mucoid colony phenotypes, which correlate with higher capsule or matrix secretion, rapidly evolve under lytic phage exposure in E. coli and Pseudomonas fluorescens (24, 25).

Beyond their deep importance to microbial natural history, the ability of phages to rapidly destroy susceptible populations makes them attractive as alternative antimicrobials (12, 26, 27). Optimizing phages for this purpose, including an understanding of phage resistance evolution among host bacteria, requires exploration of models and experiments that specifically capture the key elements of biofilm environments (28, 29). In particular, biofilm growth may have profound impacts on the relative advantages and disadvantages of phage resistance, because the spatial structure within biofilms can potentially protect susceptible cells from phage exposure (15, 17, 22, 23, 30, 31).

Here, we use high-resolution imaging of our experimental biofilm system to address this problem, exploring the population dynamics of phages, sensitive bacteria, and resistant bacteria. Our observations indicate that sensitive bacteria coexist at high densities alongside resistant bacteria because of the protection afforded by spatial structure: the resistant bacteria block phage access to sensitive cells and can even act as phage sinks. Computational studies developed in parallel to the experiments support this interpretation and indicate that the protection of sensitive cells generalizes robustly to a wide range of bacterial resistance mechanisms, baseline bacterial growth rates, phage exposure rates, and diffusivity of phages inside and outside the biofilm microenvironment.

## RESULTS

**FIG 1:**
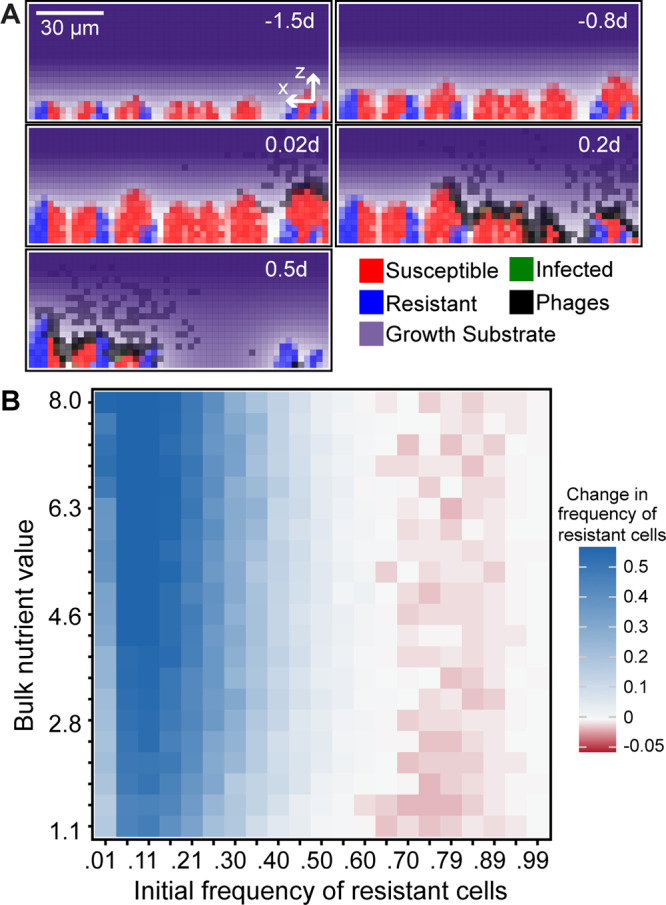
Simulated outcomes of phages exposed to biofilms composed of resistant and susceptible cells. (A) Example time series in which biofilms of phage-resistant and phage-susceptible cells are allowed to reach a critical height before introduction of phages. Phages can absorb to resistant cells but cannot amplify within them, and phages that have departed the biofilm—if they do not reinfect within the next time step—are assumed to be removed by fluid advection. Note that the colors for nutrients, bacteria, and phages are graded from light to saturated as a function of the local density of the respective class. For example, black squares contain the maximum local density of phages, whereas gray squares contain fewer than the maximum. d, day. (B) Summary heatmap of the effect of biofilm structure on selection for phage resistance. In the heatmap, simulation outcomes are shown for various degrees of nutrient availability (which controls the baseline host growth rate) and initial resistant strain frequency. Here, both phage mobility and removal rate from the liquid phase are intermediate, and the bacterial fitness cost of phage resistance is 5% of the maximum growth rate (see Fig. S1 and S2 in the supplemental material for extensive exploration of these factors). Resistant cells increase in frequency when initially uncommon (blue squares in heatmap), but when they are initially common, their relative abundance either stays the same (white squares) or decreases (red squares).

In biofilm environments, the population dynamics of bacteria and their lytic phages are driven by many processes, including bacterial growth, mechanical cell-cell shoving, solute advection/diffusion, phage-host attachment probabilities, phage lag time and burst size, and phage advection/diffusion, among others (9, 15). To study these processes, we expanded a simulation framework previously developed by our groups that captures the biological and solute/particle transport processes inherent to biofilm communities (15) (Materials and Methods; code available at https://github.com/simbiofilm/simbiofilm [32]). Our framework implements the growth of up to hundreds of thousands of discrete bacteria and phages in explicit space; it is custom-made for this application but falls within the family of biofilm simulation techniques that have been highly successful in capturing the qualitative dynamics of natural systems (33–35). To begin a simulation, cells are inoculated onto a solid surface at the bottom of a two-dimensional (2D) space with lateral periodic boundary conditions. Growth-limiting nutrients diffuse from a bulk liquid layer at the top of the 2D space toward the biofilm front, where they can be depleted due to consumption by cells (Fig. 1A). The biofilm surface erodes in a height-dependent manner, reflecting the increase in shear rate with distance from the surface (36). After a preset interval of biofilm growth, phages are introduced to the system in a pulse at one location along the biofilm’s upper surface (varying the timing or location of phage pulses had little impact on the results, see below). In the simulations, phages can associate with cells in the biofilm and initiate infections or be released into the surrounding liquid, where they diffuse for a full simulation iteration cycle prior to being swept out of the system by advection (Fig. 1A). We implemented phage diffusion by algorithmic rules that are described in detail in Materials and Methods.

10.1128/mSystems.00877-19.3 FIG S1 Parameter sensitivity analysis for predicted coexistence of phage-resistant and phage-susceptible cells. The robustness of the predictions outlined in the main text were tested with variation in the cost of phage resistance, diffusivity of phages through biofilm biomass, and speed of phage transport/removal in the liquid phase outside biofilms. As in Fig. 1 of the main text, for each parameter combination, simulations were run for a range of initial strain frequencies, and for different values of the bulk nutrient concentration, which controls the bacterial growth rate. The heatmaps depict the change in frequency of the resistant strain after phage exposure. Download FIG S1, TIF file, 1.8 MB. Copyright © 2020 Simmons et al. 2020 Simmons et al. This content is distributed under the terms of the Creative Commons Attribution 4.0 International license.

10.1128/mSystems.00877-19.4 FIG S2 The mechanism of phage resistance does not substantially impact patterns of selection for resistance in biofilms. Here, the parameter sweep analysis shown in Fig. S1 was repeated (at one quarter resolution) for three different mechanisms of phage resistance on the part of resistant hosts. On the left, phages cannot adsorb to hosts, as could be the case for mutants that have lost the phage receptor. In the center, phages can adsorb to resistant hosts and be neutralized, but the host survives the encounter and remains viable, as could be the case for CRISPR-Cas9-based resistance to phages. Finally, for comparison, on the right, we repeat the analysis of phage resistance in which both the host and phage are neutralized by the contact event. This condition represents the abortive infection resistance mechanism that is implemented in our experimental system (see the main text). Download FIG S2, TIF file, 2.7 MB. Copyright © 2020 Simmons et al. 2020 Simmons et al. This content is distributed under the terms of the Creative Commons Attribution 4.0 International license.

To understand the population dynamics of phages in the presence of biofilms that contain both susceptible and resistant bacterial strains, we constrained our simulations using experimentally measured parameters for bacterial growth, phage replication, and nutrient diffusion (see Table 1), based on E. coli and its lytic phage T7 (the same species used in our experiments, see below). We explored the impact of factors that are likely to vary in natural environments where phage-biofilm interactions occur. The first is nutrient availability, which controls overall biofilm expansion rate (37, 38). We also varied the initial population ratio of susceptible to resistant host bacteria. In this way, we could test for the invasibility of phage-resistant and phage-susceptible cells when they are rare. For example, if resistant cells always increase (or decrease) in frequency regardless of their initial fraction, we can infer that they are being positively (or negatively) selected. On the other hand, if they increase when initially rare but decrease when initially common, then we can infer that resistant and susceptible cells will tend toward coexistence (39). We also tested for the effect of variation in the fitness cost of phage resistance, variation in phage diffusivity, variation in how phages were introduced to the biofilm surface, and whether phages were introduced at earlier or later time points during biofilm growth. Importantly, we also explored the impact, if any, of the mechanism of phage resistance.

Phage resistance can manifest in different ways; for example, in the case of E. coli and the lytic phage T7, which attaches to lipopolysaccharide (LPS) to initiate infection, the host can evolve resistance by modification or partial loss of the LPS biosynthesis machinery or by loss of thioredoxin A, which is co-opted and required by T7 as a phage DNA polymerase processivity factor (40). In the case of LPS mutants, phages cannot bind the cell surface, and thus, phage-host encounters leave both phage and host intact. Mutants of E. coli that have lost thioredoxin A allow phage entry, but not replication, and thus cause an abortive infection in which the host and the phage are both killed. In our experimental tests below, we use the thioredoxin A mutant as our phage-resistant strain. Correspondingly, we use for our simulations in the main text a resistant mutant that both neutralizes phages and is neutralized when a phage attaches. However, we repeated all simulations for the scenario in which neither the host cell or phage is neutralized by a contact event and phages are free to continue diffusing and for the scenario in which the bacterial host is unaffected while the phage is neutralized by a contact event.

### Biofilms facilitate coexistence of phage-resistant and -susceptible cells.

The full results of our parameter sweeps are shown in Fig. S1 and S2 in the supplemental material, and for clarity, we show a representative subset of these results in Fig. 1B, where the fitness cost of phage resistance is a 5% reduction in the maximum growth rate, and phages are moderately impeded from diffusion in biofilms. In the absence of phage exposure, susceptible cells outcompete resistant cells more strongly as the cost of phage resistance increases (Fig. S3A). In the presence of phage exposure, the overriding pattern of our simulations was positive selection for phage-resistant cells when they are initially rare, and negative selection for resistant cells when they are initially common (Fig. 1B; Fig. S1 and S2). The exceptions occur (i) when the cost of phage resistance is zero (Fig. S1A); (ii) in some instances when phage mobility inside biofilms is high, such that the system behaves as though it were a well-mixed culture and phage resistance is uniformly positively selected (Fig. S1B-i); or (iii) when phage mobility inside and outside the biofilm is near zero, such that viral particles rarely “find” susceptible hosts by diffusion (Fig. S1A-ii, S1B-ii, and S1C-ii); in the latter case, phage-resistant and phage-susceptible cells compete according to growth rate.

10.1128/mSystems.00877-19.5 FIG S3 Control simulations for biofilm competition between phage-resistant and phage-susceptible cells as a function of the cost of phage resistance. (A) When the resistance cost is zero (bottom row), competition is neutral with drift mildly favoring whichever strain is more common at the simulation outset. For a nonzero cost of phage resistance, in the absence of phage exposure, the susceptible strain overwhelmingly outcompetes the resistant strain. When the cost is zero or when either strain is initiated in an extreme minority, the dynamics are driven by drift with the strain in the initial majority being less likely to be eliminated by random chance. (B) Illustration of bottleneck cutoff in which a susceptible cell cluster, despite having a higher growth rate and not being exposed to phages, is cut off from access to areas of sufficient nutrients to grow. This is a spatial genetic drift process along the cell group front that is more likely to eliminate rare lineages unless their growth advantage is very high. Download FIG S3, TIF file, 2.3 MB. Copyright © 2020 Simmons et al. 2020 Simmons et al. This content is distributed under the terms of the Creative Commons Attribution 4.0 International license.

An interesting peripheral note is that when resistant cells were initiated at very high frequency, selection against them was weakened (the columns in Fig. 1B above 0.99 initial resistant strain frequency are only light red). We found this to be due to a population bottleneck effect. As the biofilm front advances toward a nutrient source, cell lineages that fall behind the front are cut off from future nutrient access and extinguish; this process, by chance, tends to eliminate rare strains early during biofilm growth and so weakens the signature of selection even when that strain has a growth advantage (Fig. S3B). Overall though, this effect does not counteract selection for susceptible strains when they are rare.

We observed the same negative frequency-dependent selection for resistant cells when our simulations were implemented in 3D space (see Movies S1 and S2 in the supplemental material). The results were also the same regardless of the mode of resistance among the bacteria: the same trends were upheld if the resistant strain caused abortive infections, if neither resistant hosts nor phages were neutralized by mutual contact (as in surface-modification resistance), or if phages alone were neutralized by contact with resistant hosts (as in restriction endonuclease or CRISPR-Cas9 based resistance) (Fig. S2).

10.1128/mSystems.00877-19.1 MOVIE S1 A biofilm simulation in three dimensions illustrating the clearance effect by which susceptible cells (red) (infected cells shown in green), when common in the biofilm population relative to resistant cells (blue), are mostly or entirely killed off by a propagating phage epidemic. Download Movie S1, MOV file, 2.3 MB. Copyright © 2020 Simmons et al. 2020 Simmons et al. This content is distributed under the terms of the Creative Commons Attribution 4.0 International license.

10.1128/mSystems.00877-19.2 MOVIE S2 A biofilm simulation in three dimensions illustrating the phage sequestration effect by which susceptible cells (red) (infected cells shown in green), when initially rare in the biofilm population, are protected from phage exposure if they are surrounded by a majority of resistant cells (blue), which prevent phages from reaching susceptible hosts in which to infect and multiply. Download Movie S2, MOV file, 0.5 MB. Copyright © 2020 Simmons et al. 2020 Simmons et al. This content is distributed under the terms of the Creative Commons Attribution 4.0 International license.

The results robustly support the prediction of coexistence of phage-resistant and phage-susceptible cells in biofilm environments, provided that phage resistance carries a nonzero cost. We observed the same qualitative pattern as shown in Fig. 1B when we varied the biofilm size at which phages were introduced, and there was similarly little effect if phages were introduced at a single point or evenly along the entire biofilm surface (Fig. S4). The strength of negative frequency-dependent selection and the predicted stable frequencies of resistant and susceptible cells are tuned by phage mobility and the cost of phage resistance (15), but the overall pattern of predicted coexistence is highly robust to parameter changes when the cost of phage resistance is nonzero (Fig. S1) (39, 41–43). We also note the impact of the intensity of phage pulses into the system. The results hold when the phage pulse duration is increased by 2 orders of magnitude, but past a threshold of phage attack rate, all susceptible cells are eventually encountered by phages and selection for resistance becomes uniformly positive (Fig. S5). We next looked for the origin of negative frequency-dependence: why do phage-resistant cells fare well when rare but fare poorly when common?

10.1128/mSystems.00877-19.6 FIG S4 Extended simulations testing the robustness of negative frequency-dependent selection for phage resistance. In addition to core simulation parameters assessed in Fig. S1, we also tested for the robustness of our results against (A) variation in the threshold biofilm height at which phages were pulsed into the system, (B) whether or not resistant cell growth halts upon phage contact, which is the case for some forms of phage resistance that do not permit phage amplification but still allow phage entry into the host cell, and (C) whether phages were introduced in an even layer across the biofilm upper surface, or at a single point on the biofilm surface. All other parameters in these simulations are the same as those used for simulations summarized in Fig. 1 of the main text. Download FIG S4, TIF file, 1.8 MB. Copyright © 2020 Simmons et al. 2020 Simmons et al. This content is distributed under the terms of the Creative Commons Attribution 4.0 International license.

10.1128/mSystems.00877-19.7 FIG S5 Variable phage pulse duration and the effect on selection for phage resistance. The negative frequency-dependent selection pattern is upheld even for pulse durations that are an order of magnitude longer than those used in the analysis in the main text. However, in the limit of constant phage exposure, most or all susceptible cells are killed, and selection for phage resistance becomes uniformly positive. Download FIG S5, TIF file, 1.8 MB. Copyright © 2020 Simmons et al. 2020 Simmons et al. This content is distributed under the terms of the Creative Commons Attribution 4.0 International license.

**FIG 2:**
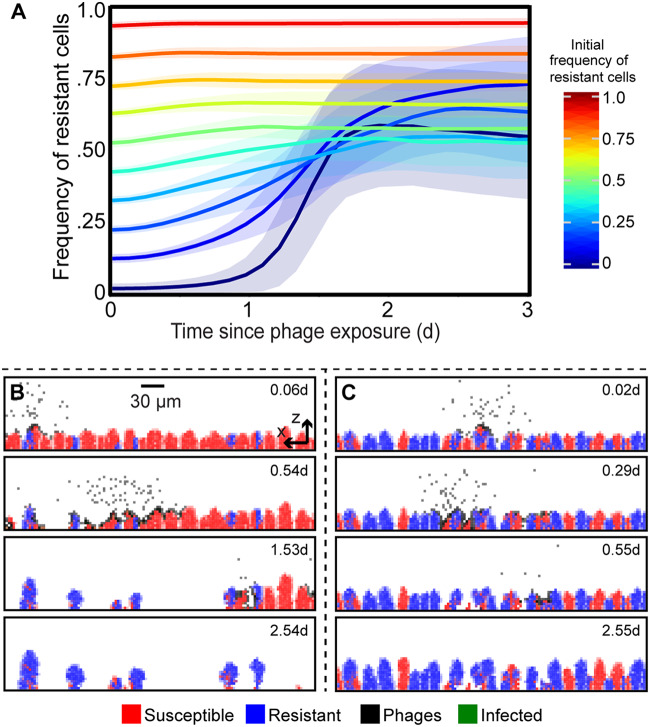
Simulated population dynamics of phage-resistant and -susceptible bacteria within biofilms. These dynamics underlie the competition outcomes in Fig. 1. Time is shown in days (d). (A) The frequency of resistant cells is shown in traces colored according to their initial frequency, with the standard deviation across all replicate runs as transparent blue regions around each trace (*n *= 90 to 100 replicate simulations per trace). (B) When resistant cells are initially a minority, susceptible cells are exposed to phages and largely killed off, allowing resistant cells to reseed the population and markedly increase in relative abundance relative to the strain ratio prior to phage exposure. (C) When resistant cells are initially more common, and phages cannot diffuse freely through the biofilm, susceptible cells are spatially protected from phage exposure because phages are sequestered in clusters of resistant cells.

**(i) Clearance of susceptible cells when they are common.** When phage-susceptible cells start in the majority within a biofilm, the few resistant cells initially in the population are concentrated into small isolated groups. As a result, when phages enter the system, they have ready access to susceptible hosts that occupy the majority of space, and the propagating infection eliminates most or all of the susceptible population. After this clearance event, the few remaining phage-resistant cells have an abundance of open space to occupy as they continue to grow with reduced competition for nutrient sources in the surrounding medium (Fig. 2A and B). Unless the cost of phage resistance is zero (Fig. S1A), resistant cells tend not to reach fixation due to small pockets of susceptible cells that are protected from phage exposure by neighboring resistant cells (Fig. 2B). The latter effect is strengthened if resistant cells are initially abundant, as detailed below.

**(ii) Phage sequestering by resistant cells when they are common**. When phage-resistant cells are initially common, phage-susceptible cell clusters are isolated among larger groups of resistant cells. If phage diffusion is even moderately impeded by the presence of biofilm, then susceptible cells gain protection from phages. This occurs because phages become trapped on the periphery of clusters of resistant cells and because phages released into the liquid phase are often blocked from long-range movement by groups of resistant cells in their path. The lower the frequency of susceptible cells in the initial inoculum, the stronger the effect of these spatial phage protection mechanisms. In this scenario, if there is no cost to resistance, then susceptible and resistant cells compete neutrally. If there is a fitness cost to resistance, then susceptible cells have an intrinsic growth rate advantage, and they increase in frequency if they are initially rare (Fig. 2A and C).

### Experimental model of phage resistance population dynamics.

Our simulation results predict coexistence of phage-susceptible and phage-resistant cells in biofilm environments, nearly regardless of variation in any major features of the system. Here, we set out to test this prediction using an experimental model of biofilm growth under lytic phage attack. Biofilms of E. coli were cultivated in microfluidic devices, including cocultures of wild-type (WT) strain AR3110, which is T7 susceptible, and an isogenic strain harboring a clean deletion of *trxA*, which does not support phage replication (see Materials and Methods). The Δ*trxA* mutant lacks thioredoxin A, which is an essential DNA processivity factor for the lytic phage T7. This deletion mutant therefore causes an abortive infection in which phage attachment occurs and the host is killed, but the phage is not able to replicate or lyse the host (40).

We chose the Δ*trxA* mutant as representative of phage-resistant variants because it does not support phage propagation but is able to form biofilms normally. Almost all other mutations conferring T7 resistance are in the LPS assembly machinery (40), and our pilot experiments indicated that these mutant classes are defective for biofilm formation and so do not allow the experiments described below to be performed. This biofilm defect is a notable fitness cost of LPS modification-dependent phage resistance, as the ability to form matrix-enclosed biofilms is increasingly accepted as a critical currency of fitness in many natural environments (44). This suggests that even though there are LPS mutations that confer phage resistance, they may often be selected against in many natural environments because they impair biofilm formation or other important surface interaction phenotypes. Many other known resistance mechanisms, such as restriction endonuclease production, CRISPR-Cas9 adaptive immunity, and an expanding list of others (45), should not suffer the fitness cost of biofilm deficiency, because they are mechanistically uncorrelated with biofilm production.

To test our predictions, we required a T7-resistant mutant capable of biofilm formation and so focus on the Δ*trxA* background for the remainder of the paper. Growth curves in shaken liquid media identical to that used for biofilm experiments indicated that the phage-resistant Δ*trxA* mutant has a growth rate cost of 7.9% ± 0.69% compared with the WT (Fig. S6A). The E. coli experimental biofilms were cultivated in microfluidic flow devices composed of a chamber molded into polydimethylsiloxane (PDMS), which was then bonded to a glass coverslip for imaging on an inverted confocal microscope. The biofilm images were analyzed and quantified with the BiofilmQ software (46). Prior work has shown that even biofilms of phage-susceptible WT E. coli AR3110 can protect themselves from phages after ∼60 h of growth, when they begin to produce a curli amyloid fiber mesh, which is primarily located on the outer surface of the biofilm and blocks phage diffusion (22). Here, biofilms of WT and Δ*trxA* mutant were cultivated for only 48 h prior to phage exposure, such that no curli-mediated phage protection could occur during the initial phage exposure.

10.1128/mSystems.00877-19.8 FIG S6 Characterization of phage-resistant and phage-susceptible strains of E. coli used in our experiments. (A) Growth curves of E. coli wild-type AR3110 (phage T7 susceptible [blue]) and Δ*trxA* mutants (phage T7 resistant [red]) in tryptone broth liquid culture with shaking at 30°C. Data points denote mean values of six independent runs of the experiment. Standard deviations are shown but are smaller than the data point symbols. Fitting each run to the logistic growth equation yielded an average maximum growth rate of 0.40 ± 0.004 h^−1^ for the phage-susceptible WT, and a maximum growth rate of 0.37 ± 0.002 h^−1^ for the phage-resistant Δ*trxA* mutant. (B) WT biofilms after 48-h growth are susceptible to T7 phage attack and are killed after phage exposure. Any remaining cells show a green fluorescent protein (GFP) signature of T7 infection from the *sfGFP* locus encoded on the genome of the T7 phage used here. (C) *trxA* deletion mutant biofilms are morphologically identical to WT biofilms at 48 h but show minimal disruption by an equivalent pulse of T7 phages. Download FIG S6, TIF file, 2.2 MB. Copyright © 2020 Simmons et al. 2020 Simmons et al. This content is distributed under the terms of the Creative Commons Attribution 4.0 International license.

We used an engineered strain of phage T7 encoding on its genome a copy of *sfGFP* under the control of a strong constitutive promoter; this strain has been extensively verified in prior work (22). The construct causes host cells, when infected, to fluoresce green (in addition to the host’s constitutive fluorescent color), prior to lysis. We confirmed that our wild-type E. coli AR3110 biofilms were killed off by T7, while biofilms of the resistant *trxA* mutant were not (Fig. S6B). In different runs of the experiment, mimicking our simulation approach, we inoculated the glass bottom of flow devices with various ratios of phage-susceptible and phage-resistant bacterial cells. We allowed biofilms to grow undisturbed for 48 h and then subjected them to a pulse of high-density phage suspensions (Fig. S7). Biofilm populations were then imaged by confocal microscopy at regular intervals for 2 days. For each imaging session, the entire biofilm volume was captured in successive optical sections.

10.1128/mSystems.00877-19.9 FIG S7 Diagram of experimental biofilm growth and phage treatment regime. (A) Biofilms were grown by inoculating phage-susceptible and -resistant cells in controlled ratios (see main text) onto the glass bottom of PDMS microfluidic devices. (B) Biofilms were grown in the absence of phage for 48 h, after which (C) the medium inlet tubing was switched to perfuse biofilms with T7 phages. (D) The inlet tubing was replaced again to continue flow of fresh media, and image series were acquired by confocal microscopy. Download FIG S7, TIF file, 0.4 MB. Copyright © 2020 Simmons et al. 2020 Simmons et al. This content is distributed under the terms of the Creative Commons Attribution 4.0 International license.

**FIG 3:**
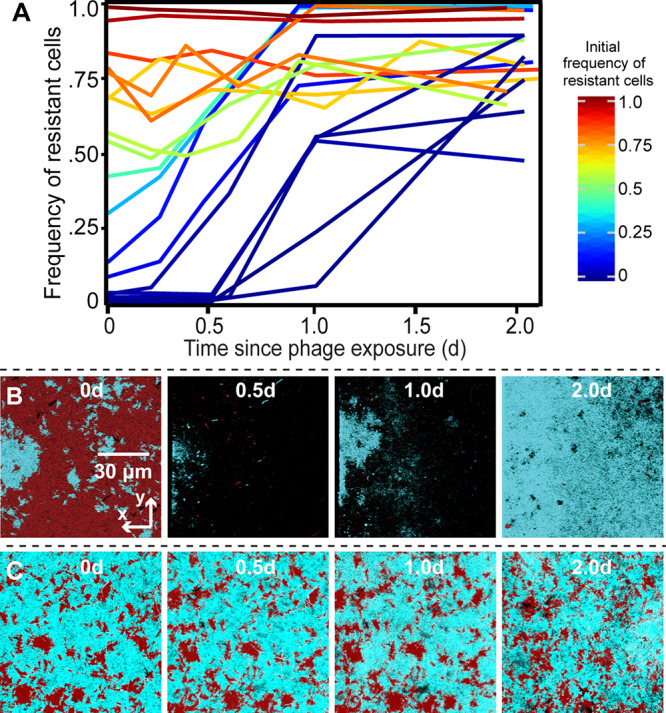
Experimental test of model predictions for phage-biofilm coexistence. Biofilms containing mixtures of phage T7-susceptible E. coli AR3110 and a phage T7-resistant mutant carrying a deletion of *trxA* were grown for 48 h before administering a pulse of phages to the two-strain biofilm population. The frequency of resistant cells is shown in traces colored according to their initial frequency, where each trace is an independent run of the experiment. (A) Population dynamics traces showing the frequency of phage-resistant E. coli as a function of its initial population frequency. Each trace is a single replicate of the experiment, with various initial ratios of the two strains as in our simulations. (B and C) Time series of phage-resistant (blue) and phage-susceptible cells (red) following a pulse of phages into the chambers. The panels from left to right show biofilms at ∼0, 0.5, 1, and 2 days after phage exposure. Each image is an *x*-*y* optical section from a stack of images covering the whole biofilm volume, acquired by confocal microscopy.

**FIG 4:**
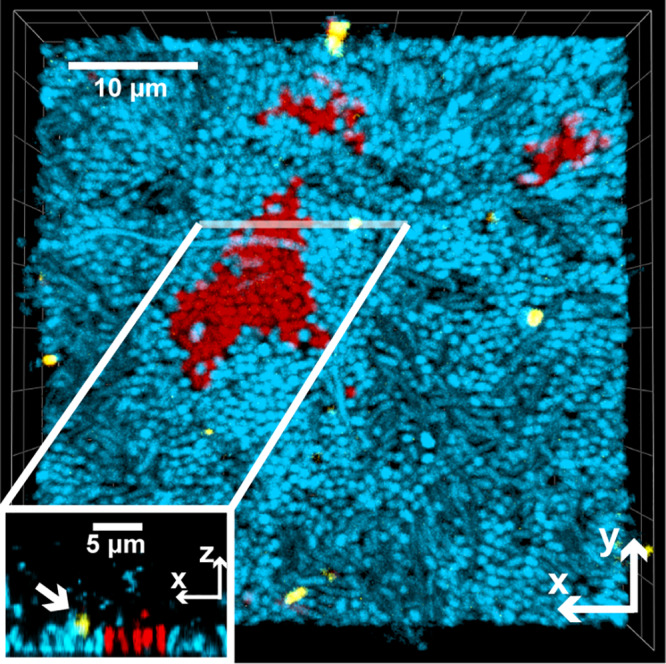
Experimental demonstration of phage sequestration within clusters of phage-resistant bacteria (blue) in a mixed-strain biofilm with phage-susceptible bacteria (red). Purified phages stained with Alexa Fluor 633 (shown in yellow) were added to biofilms grown for 48 h in which resistant cells were inoculated as 95% of the founding population. The central image is a top-down view of a 3D rendering measuring 50 μm × 50 μm × 15 μm (length × width × depth). The inset image is a 2D projection of a vertical slice through a 3D volume at the indicated location. The white arrow in the inset points to an immobilized phage on a cluster of resistant cells. Note that phages are much smaller than the minimum resolvable volume of a confocal fluorescence microscope like the one used here; as a result of this optical effect, the phages appear larger than their true size.

We found that when phage-resistant cells were initially rare, susceptible cells were killed off by phage exposure and mostly cleared out of the chambers, opening new space into which resistant cells could grow for the remainder of the experiment (Fig. 3A and B). As in our simulations, resistant cells often did not reach fixation, as small clusters of susceptible cells remained. On the other hand, when phage-resistant cells were initially common (∼60% of the population or more), the relative fraction of resistant and susceptible host bacteria did not substantially change following phage treatment (Fig. 3A and C). Control experiments confirmed that evolution of resistance among the originally susceptible population could not explain these results, as there were on average fewer than 15 *de novo* resistant mutants across the entire area of the chamber devices, which contained on average 3.8 × 10^6^ cells at the time of phage exposure (as measured in four replicate chambers; see Materials and Methods). Rather, the susceptible cells were protected from phage exposure due to immobilization of phages in clusters of resistant cells in the vicinity, which we could confirm by introducing fluorescently labeled phages to mixed biofilms of resistant and susceptible cells (Fig. 4 and Fig. S8). This method labels just one generation of phages, but these data demonstrate that phages can be immobilized within clusters of resistant cells, by adsorption to the cells themselves or by entrapment in the milieu between resistant cells. The susceptible cells were not entirely invulnerable when rare; we could document regions where we interpret patches of susceptible cells had been cleared by an infection event (Fig. S8). In this sense, we would predict that the protection of susceptible cells scales with the intensity of phage exposure and the relative abundance of resistant cells in the population. We tested this idea in our simulations, finding that if phage exposure is continuous for sufficiently long periods, all susceptible cells are killed (Fig. S6).

10.1128/mSystems.00877-19.10 FIG S8 Phages (yellow) trapped by majority resistant bacteria (blue) are unable to reach and infect sparse patches of susceptible cells (red). (A to H) Additional replicates of the experiment depicted in Fig. 4 of the main text, in which biofilms inoculated with 20:1 resistant-susceptible cells were grown for 48 h and then pulsed with phages for prior to imaging by confocal microscopy. Each panel above is a 3D biofilm volume rendering ∼50 μm × 50 μm × 15 μm (L × W × D). Note that the top left panel is a recapitulation of Fig. 4 for comparison with other samples. In the case of panel G, we speculate that we captured an event in which one patch of susceptible cells was killed off by a phage encounter (indicated by white arrows). Download FIG S8, TIF file, 2.3 MB. Copyright © 2020 Simmons et al. 2020 Simmons et al. This content is distributed under the terms of the Creative Commons Attribution 4.0 International license.

Our experimental results thus displayed a good qualitative match to our simulation models. The spatial patterns underlying these outcomes were comparable to those observed in our simulations, including a clearance of susceptible cells when resistant cells are initially rare. In this condition, susceptible cells are exposed to phages; the remaining resistant cell clusters then have ample room to multiply. Our experiments also confirmed that susceptible cells are often protected when they are initially rare: when resistant cells are common, they often sequester phages away from susceptible cells, which then remain near their initial frequency in the population.

## DISCUSSION

Our results provide a theoretical and empirical understanding of how decreased phage mobility among resistant hosts influences the population dynamics of phage resistance within biofilms. Using simulations with extensive parameter sweeps, we found a dominating trend toward negative frequency-dependent selection for phage resistance that is robust to parameter changes. This outcome is matched by our microfluidic model of biofilm formation, and it reinforces and generalizes predictions from abstract models in the literature (14, 16, 29).

The origins of frequency-dependent selection are tied to the cell movement constraints and competition for space in biofilms. When phage-resistant bacteria are initially rare, introduced phages have open access to susceptible hosts, which are mostly killed, leaving empty space for the residual resistant cell clusters to occupy. On the other hand, when phage-resistant bacteria are initially common, they create barriers between phages and clusters of susceptible cells. So long as there is impeded diffusion of phages through the biofilm volume, this spatial arrangement provides protection to susceptible cells, whose population frequency then drifts or increases depending on the fitness costs of phage resistance. On the basis of our parameter sensitivity analyses, we infer that this pattern is an inevitable consequence of the spatial constraints inherent to biofilm communities.

We tested these outcomes experimentally using microfluidic culture and confocal microscopy of mixed E. coli biofilms containing resistant and susceptible hosts; these trials gave a good qualitative agreement with the simulations. We could document both the clearance and phage immobilization effects, depending, as anticipated from simulations, on the initial fractions of resistant and susceptible bacterial cells. Because LPS mutants of E. coli appear to be impaired for biofilm formation, we were only able to experimentally test the case in which abortive infection is the resistance mechanism. In this manner, phages are limited in their diffusion not only because of barriers of resistant cells but also because of sorptive scavenging; that is, they are sequestered by resistant cells, and both the host and phage are neutralized by the encounter (16, 47–51). We emphasize, however, that our simulations strongly suggest that the same pattern of negative frequency-dependent selection for resistance will be upheld regardless of the resistance mechanism.

Our results also draw an analogy between phage “epidemics” on the submillimeter scale of biofilms and the process of herd immunity studied for decades at much larger spatial scales in populations of plants and animals (52–54). When enough of the population is resistant, a spreading pathogen is no longer able to establish sufficient infections to amplify itself, and the susceptible portion of the population is protected (52). These observations in turn have several general implications. We anticipate that the arms race of phage attack and host defense can have a quite different landscape in biofilms compared with planktonic populations (2, 5, 7, 14, 55). A rich history of research has shown that phages can rapidly eliminate susceptible host cell populations in mixed liquid culture (2–4), though this is not always the case, depending on the resistance mechanism and relative abundance of phages and hosts (56, 57). In biofilms, by contrast, our results predict widespread and easily maintained polymorphism in phage resistance ability. This kind of standing variation can also arise due to minority advantage (i.e., kill-the-winner) mechanisms (58–61), in which phages or other parasites are selected to target the most abundant constituent strains of a population.

The mechanism we describe here is distinct from kill-the-winner-based selection, but complementary: susceptible cells in the minority are unlikely to be exposed to phages in the first place, as they are shielded by resistant cells blocking phage diffusion. Coevolution between phages and host bacteria, therefore, is likely to take different evolutionary trajectories that move at slower speeds than those typically observed in shaken liquid culture test tubes. This outcome echoes results observed in the early phage-host coevolution literature, where it found that for bacteria that form “wall populations” on the inside of shaken liquid culture tubes, phage-susceptible bacteria survive at much higher rates than in the well-mixed planktonic phase (62). These wall populations are now known as biofilms, and here we have directly visualized the spatial protection process that allows susceptible cells to survive where otherwise they would not. The results obtained here also make concrete on the microscopic scale how variable access of phages to susceptible hosts shifts populations to steady states in which phage-resistant and phage-susceptible bacteria should robustly coexist (5, 14).

Our observations also bear on the efficacy of phage therapies, for which one of the most promising potential benefits is selective elimination of target pathogens from a community of otherwise commensal or beneficial microbes (12, 27, 60, 63, 64). This could potentially be an advantage relative to broad-spectrum antibiotics that can kill off not just the target pathogen but also many other members of a patient’s microbiota, sometimes with severe side effects (65). Our work suggests that phage-susceptible cells can be much harder to target and can coexist with resistant cells, or presumably cells of other species that phages cannot target, due to the protective effects of phage diffusion blocking. Successful phage treatment will likely depend on disruption of the biofilm architecture to ensure exposure of target bacteria to the therapeutic. It should be noted, however, that our work here examines only two strains of the same species, and whether these conclusions apply to multispecies consortia (66), whose biofilm architectures can differ substantially, is an important topic for further work.

The models developed here do not address the possibility of refuges created by nutrient-starved bacteria in the basal layers of biofilms where nutrients have been depleted. This did not appear to be an important feature of our experimental biofilms, which agreed well with simulation predictions. However, quiescent cells could potentially be significant in other conditions, especially for cell groups that accumulate thicker mats with large, nutrient-starved populations in their interior (67). We also do not implement ongoing mutations in the different bacterial and phage strains residing in biofilms, using instead strains that are fixed in either the phage-susceptible or -resistant state to examine short-term population dynamics. Last, and importantly, we omitted from our study the possibility of temperate phage infections, in which the phage genome is inserted into that of the host organism. Temperate phages present a wide diversity of potential outcomes, especially considering that they can impart new phenotypes to their bacterial hosts. Tackling the challenge, both theoretically and experimentally, of how temperate phages enter, alter, and evolve within multispecies microbial communities is an important area for future work.

## MATERIALS AND METHODS

### Phage-biofilm modeling simulation framework.

The simulation framework used for this study is an updated and expanded version of a modeling approach developed by Simmons et al. (15). The major changes include a new implementation of bacteria as individual particles, rather than a homogeneous biomass, and a new implementation of phage diffusion, detailed below. The simulations are built on a grid-based approach for tracking bacteria, phages, and solute concentrations; spatial structure in the system is thus resolved at the level of grid nodes (which are 3 μm by 3 μm for the simulations described in this paper). Within a grid node, bacteria and phages are tracked individually but assumed to interact randomly. Using the FiPy partial differential equation solver for Python (68), the same grid system is used to calculate nutrient diffusion from a bulk layer above the biofilm toward the cell group surface, where it is consumed by bacteria (35, 37, 69).

As a result of nutrient consumption on the biofilm’s advancing front (Fig. 1A), nutrient gradients are created with high nutrient availability in the outer cell layers and lower nutrient availability with increasing depth into the biofilm interior. Cells near the liquid interface grow with the maximum growth rate, while cells deeper in the biofilm interior grow relatively slowly. Fluid flow is modeled implicitly; following prior literature, we allow the biofilm to erode along its outer front at a rate proportional to the square of the distance from the basal substratum (described in detail by Simmons et al. [15]). Further, any phages that depart from the biofilm into the surrounding liquid are advected out of the simulation space within one iteration cycle, which is approximately 7 to 8 min in simulation time (see below).

The simulation framework was written in an object-oriented style. A simulation object is defined via the space of the system, number and properties of implemented grid node containers, biological behaviors of bacteria and phages, one-time events (e.g., phage pulse), and simulation exit conditions. Briefly, the space of the system specifies physical information such as physical size and length scale of the grid node array in which cells, phages, and solutes are implemented. Programmatic “containers” hold the information about each modeled individual present in the system. Behaviors describe a container’s interactions with anything else, including other containers, space, or time. Events are one-time-use and include the inoculation of the system with bacteria or pulses of phages into the simulation space.

Simulations were initiated by first defining the types of container contents, including both bacterial strains/species of interest (phage susceptible and phage resistant), phage-infected bacteria, phages, and the growth substrate as a solute. This process includes specifying values for basic biological and physical parameters in the system (e.g., bacterial growth rate, phage infection rate per host-virus contact, phage lag time, phage burst size, nutrient diffusivity, and others; the full list of parameter values and their measurement origins is provided in Table 1). After containers are established in each simulation instance, the simulation proceeds through inoculation of the two bacterial species on the substratum. Phages were not introduced at the outset of simulations but rather at a set time after bacteria were permitted to grow, as described in the main text. Simulations proceed along the following cycle of steps:

1. diffusion of the nutrient substrate
2. biomass growth and division
3. lysis of infected bacteria, phage burst
4. erosion of biomass
5. phage movement
6. detachment of biomass
7. phage infection
8. biofilm relaxation (“shoving”)
9. detachment of bacteriophage

**TABLE 1:** Model parameters used for simulations

Parameter	Value(s) used in the simulations	Description	Reference(s) where applicable^*a*^	Representative value ranges and additional references, where applicable
*x*_max_,*y*_max_	900 μm, 150 μm	Physical size of the system	N/A	
*dl*, *dV*	3 μm, 27 μm^4^	Length and volume of a grid element	N/A	
*N*_max_	1.1–8 mg liter^−1^	Maximum density of substrates (range of values investigated in this study)	83	
*N*_max_	0.055−0.4 mg liter^−1^	Well-mixed simulation nutrient availability	84	
*D_N_*	2.3 × 10^−6^ cm^2^ s^−1^	Diffusivity of substrate	83	
*h*	15 μm	Diffusion boundary layer height		
*K_N_*	1.18 mg liter^−1^	Half-saturation constant for substrate	35, 85	5−225 for biofilm heterotrophic bacterial biomass, including fecal coliforms, e.g., E. coli (85, 86)
				4.86 for Pseudomonas putida F1 on glucose (87)
δ*_E_*	20 (m h)^−1^	Erosion constant	36	
*m_s_*	10^−12^ g	Bacterial mass per cell	88	10^−12^ for E. coli DSM 613
*μ_s_*^*b*^	14.1 day^−1^	Maximum growth rate	89	17.8 for E. coli K-12 on glucose (90)
				4.8−17.6 for E. coli K-12 on different substrates (91)
				6.1 for wastewater heterotrophic bacterial biomass (92)
*S*_max_	200 g liter^−1^	Maximum active biomass density	93	
*Y*	0.495	Yield of substrate converted to biomass	74	0.69–0.77 for wastewater bacteria (94)
				0.41 for E. coli K-12 on glucose (90)
				0.41−0.51 for P. putida F1 on glucose (87)
*β*	120	Phage burst size	8, 95	Bacteriophage T7
*D_P_*	3.82 × 10^−7^ cm^2^ s^−1^	Phage diffusivity constant	This study	Bacteriophage T7
*I*	0.067−0.12 (*m_s_* μm^3^)^−1^ s^−1^	Rate of interaction of phage particles with biomass particles	This study	
*δ_P_*	0.001−10 (μm^2^ h)^−1^	Phage removal rate	8, 95	
*τ*	28.8 min	Incubation period before lysis	15	Bacteriophage T7
*γ*	2.92 h^−1^	Infection rate per biomass per phage		

a N/A, not available.

b The maximum growth rate is determined from the model equations as *μ_J_* = *q_J_Y*. *q_J_* is the substrate uptake rate with a value of 28.5 g day^−1^ as in Lapisdou and Rittman (89).

The relative position of most steps is biologically motivated. The phage-related events, for example (lysis/burst, diffusion, and infection of new hosts), are in sequence relative to each other as motivated by the life cycle steps of a lytic phage. Phage detachment is placed last in the cycle so that phages may be visualized before a fraction of them is swept out of the system as described above. The second set of steps constrained with respect to each other are the biofilm dynamics: diffusion of nutrients, biomass growth, cell division, and detachment; these again follow the sequence with which cells obtain growth substrate, grow, divide, and then can be lost from the system due to detachment. Updating substrate concentrations as a function of the local bacterial biomass density, as estimated at the previous iteration, is consistent with a large body of literature on individual-based modeling for bacterial biofilms (15, 34, 35, 70, 71). Consequently, the ordering of shoving step is the only behavior that is “free floating.” We place it at the end of the biofilm dynamics sequence primarily by historical precedent in the field of biofilm spatial modeling (34, 35).

### Phage mobility and infection.

All processes describing phage-bacterium dynamics are equivalent to those presented by Simmons et al. (15) with one exception pertaining to the methods of computing phage entry and exit from the biofilm bacterial volume. This new approach is described in detail below.

Previously, we analytically solved the diffusion equation to approximate the phage density as a function of location in the biofilm. Here, in order to accommodate for possible biological heterogeneity in bacteriophage dynamics (72, 73), we introduced an algorithm for calculating phage movement by modeling each phage’s individual Brownian motion as a random walk. To account for the effect of the biofilm matrix on phage movement, we introduced a new model parameter (the interaction rate, *I*) controlling the diffusivity of phages through areas of simulation space occupied by bacterial biomass (15). We also introduce a rate of removal ($\delta_{p}$) which accounts for the removal of the phage due to the advection of the system during the phage’s motion through the space off the biofilm, scaling with the square of the distance away from the biofilm. There is an additional implicit advective removal of bacteriophage at the end of the iteration (step 9 above) where any phages remaining off the biofilm are removed from the space via advection.

The improved implementation of phage mobility operates as follows. For each phage, we first calculate the number of potential steps that could be taken in the next time interval as $n=D_{p}\mathrm{dt}/2dl^{2}$ and the time of these steps as $dt_{p}=2dl^{2}/D_{p}$, where *dl* is the grid length scale, *D_p_* is the diffusivity of the phage, and *dt* is the simulation time step. Next for each step in *n*, (i) if the phage virion is off the biofilm, determine whether the phage is removed from advection with probability $p=1-e^{dt_{p}d^{2}\delta_{p}}$, where *d* is the distance away from the biofilm. (ii) Next choose a target node by randomly choosing direction. The probability to remain in the current grid node depends on the number of dimensions (see calculation of phage diffusion properties below). (iii) Determine whether the phage is able to diffuse into the target grid location with probability $p=1-e^{dt_{p}(I_{s}+I_{t})}$, where *I_t_* is the interaction rate at the target grid node, and *I_s_* the interaction rate at the source node. The interaction rates are given by $I_{x}=\underset{i}{\sum }m_{x}^{i}I_{x}^{i}$ summing over the product of the rate parameter and the biomass of each individual i to get the rate at node x. (iv) Finally, if the phage has interacted with biomass, cease motion. If it has not, move the phage to the target grid node. As the interaction rate, *I*, increases, the ability of the phage to diffuse through biomass decreases (e.g., *p* tends to 1), which is a per-individual-phage representation of the phage impedance parameter previously described by Simmons et al. (15). Once the phage stops moving, we evaluate the remaining time as $dt_{r}=(\mathrm{dt}\times s)/n$, where *s* is the number of steps taken, from 0 to *n*, and use it in the infection step. To check for infection, we first randomly select a biomass particle in the grid node of the phage, weighted by the interaction rate of the individual (*m^i^ I^i^*). The particle (if susceptible) is then infected with probability $p=1-e^{-dt_{r}\text{γ}}$, where γ is the rate of infection of the phage particle. If enabled, the phage is removed on “infection” of resistant bacteria as well.

### Calculation of phage diffusion properties.

The model for an individual phage taking a step across the grid nodes is that it must diffuse a large enough distance from a grid node. The unnormalized probability density of diffusing in one place can be described by the solution of the diffusion equation in radial coordinates: $e^{-r^{2}/(adt_{p}D_{p})}$. Here, r indicates the distance away from the starting point, a is a constant indicating dimension: a=1 for two dimensions and a=4 for three dimensions, while other terms are explained above. To obtain the probability of remaining in a radius ρ, we integrate from 0→ρ over r with a normalization factor which is an integration over all space ($\int_{0}^{\infty }e^{{-r}^{2}/\left(a\;{dt}_{p}D_{p}\right)}$). Letting $\text{ρ}=\frac{\mathrm{dl}}{\sqrt{\text{π}}}$ gives a circle whose area is equal to the area of a grid node, and noting that $dt_{p}=2dl^{2}/D_{p}$, the integration yields $\text{erf}(\frac{1}{\sqrt{2a\text{π}}})$, or p=0.42 in two dimensions and p=0.22 in three dimensions.

### Details on simulation initial conditions and execution of parameter sweeps.

Where possible, biological and physical parameters of the simulation system were constrained according to experimentally measured values for E. coli and phage T7, which were the focal species of our experiments as well (Table 1). Following our previous biofilm dynamics simulation work (15, 38, 74), each simulation starts with an initial ratio of phage-susceptible and -resistant strains on the solid substratum, and these two strains compete for access to space and growth-limiting nutrients that diffuse from a bulk layer above the biofilm. When the biofilm height reaches 30 μm (approximately 7 days for the lowest condition and 1 day for the highest), there is a pulse of bacteriophages to the highest point of susceptible biomass, simulating an individual cell bursting, releasing bacteriophages. Repeating our simulation parameter sweeps with earlier (20-μm biofilm height) or later (50-μm biofilm height) phage inoculation had no effect on the qualitative outcomes (Fig. S4A). Two phage inoculation methods were tested. The first approach to phage inoculation was a 120-virion pulse at a single position at the highest point of susceptible biomass in the biofilm. The second was a “spray” of phages in the area just above the biofilm outer surface: 300 phages are added to randomly selected grid nodes 9 μm above the biofilm. No qualitative differences between these methods was found (Fig. S4C). Data reported in Fig. 1 correspond to simulations obtained using the first method, but we confirmed that the core results are upheld when using the “spray” method of phage inoculation.

Simulations were run until one of two different exit conditions was reached: either susceptible or resistant cells going to fixation in the population, or the simulation ran to a prespecified endpoint (time of infection + 10 days). Simulations were run for 21 different nutrient bulk values corresponding to an approximate time of infection at 1 through 7 days, where the faster growth has slightly greater strain mixing (75, 76). The initial resistant strain frequency also varied from 1% to 99% in 21 steps. Additional simulations were run also for three distinct fitness cost levels of phage resistance and for three different values of the interaction rate parameter *I*, which effectively varied phage mobility through biofilms easily penetrating the biofilm surface to severely impeded immediately upon biofilm contact (see main text). We ran 100 simulations with different random seeds to completion for each combination of parameters in the main text. For the phage pulse event in each simulation, 120 discrete phage particles (i.e., the number of phages corresponding to one burst) were added into the system with a single pulse. We wanted to examine cases in which at least one initial phage infection occurred, as otherwise a given simulation would be the same as a null control without the addition of phages. There was certainly no maximum phage population size; rather the 150-phage threshold was used to trim simulations in which the addition of phages resulted in zero infections. We inspected these data carefully and found no particular bias in terms of simulations in which this occurred. On average, this resulted in ∼90 replicate simulations per parameter combination in each of the heatmaps shown in the main text and supplemental figures.

### Experiments.

**(i) Bacterial strains.** Both strains used in this study are E. coli AR3110 derivatives, created using the lambda red method for chromosomal modification (77). The Δ*trxA* deletion strain was created by amplifying the locus encoding chloramphenicol acetyltransferase (*cat*) flanked by FRT recombinase sites, using primers with 20-bp sequences immediately upstream and downstream of the native *trxA* locus. The FRT recombinase encoded on pCP20 was used to remove the *cat* resistance marker after PCR and sequencing confirmed proper deletion of *trxA*. The wild-type E. coli AR3110 was engineered to constitutively express the fluorescent protein mKate2, and the *trxA* null mutant was engineered to constitutively produce the fluorescent protein mKO-κ. These fluorescent protein expression constructs were integrated in single copy to the *attB* locus on the chromosome, and they allowed us to visualize the two strains and distinguish them in biofilm coculture by confocal microscopy.

**(ii) Biofilm growth in microfluidic channels.** Microfluidic devices were constructed by bonding polydimethylsiloxane (PDMS) castings to cover glass of thickness number 1.5 and size 36 mm by 60 mm (ThermoFisher, Waltham, MA) (78, 79). Bacterial strains were grown in 5 ml lysogeny broth (10 g tryptone, 10 g NaCl, 5 g yeast extract liter^−1^) overnight at 37°C with shaking at 250 rpm. In the morning, cells were back-diluted into tryptone broth (10 g liter^−1^ tryptone) and then harvested at mid-exponential phase (optical density at 600 nm [OD_600_] = 0.4 to 0.5). Cells were pelleted and washed twice with 0.9% NaCl before normalizing to OD_600_ = 0.2. Strains were combined in various ratios (see main text) and inoculated into channels of the microfluidic devices. Bacteria were allowed to colonize for 1 h at room temperature (21 to 24°C) before providing constant flow (0.1 μl/min) of tryptone broth. Medium flow was achieved using syringe pumps (Pico Plus Elite, Harvard Apparatus) and 1-ml syringes (25-gauge needle) fitted with number 30 Cole-Palmer PTFE tubing (inner diameter [ID] = 0.3 mm). Tubing was inserted into holes bored in the PDMS with a catheter punch driver.

**(iii) Bacteriophage amplification and purification.** T7 phages (22) were used for all experiments. E. coli AR3110 was used as the phage host for amplification. Purification was conducted according to a protocol developed by Bonilla et al. (80). Briefly, overnight cultures of E. coli AR3110 were back-diluted 1:10 into 100 ml lysogeny broth supplemented with 0.001 M CaCl_2_ and MgCl_2_, and incubated for 1 h at 37°C with shaking; phages from a frozen stock were inoculated and incubated until the culture cleared completely as assessed visually. Cultures were pelleted, sterile filtered, and treated with chloroform. Chloroform was separated from lysate via centrifugation and aspiration of supernatant. Phage lysate was then concentrated and cleaned using phosphate-buffered saline and repeated spin cycles of an Amicon ultra centrifugal filter units with an Ultracel 200-kDa membrane (Millipore Sigma, Burlington, MA). Purified phages were stored at 4°C.

**(iv) Bacteriophage labeling.** Phage labeling began with a high-titer phage prep (2 × 10^10^ PFU/ml) produced using the method described above. Nine hundred microliters of the phage suspension was combined with 90 μl sodium bicarbonate (1 M, pH 9.0) and 10 μl (1 mg/ml) amine-reactive Alexa Fluor 633 probe (ThermoFisher, Waltham, MA) and incubated at room temperature for 1 h. In this manner, the phages were conjugated to dye nonspecifically at one or more locations on their capsid coats. Labeled phage were then dialyzed against 1 liter of phosphate-buffered saline to remove excess dye using a Float-A-Lyzer G2 dialysis device with a molecular weight cutoff (MWCO) of 20 kDa (Spectrum Labs, Rancho Dominguez, CA). Labeled phage were diluted in tryptone broth (10 g liter^−1^ tryptone) to working concentration (2 × 10^7^ PFU/ml) prior to use.

**(v) Phage-biofilm microfluidic experiments.** Biofilms consisting of various ratios of susceptible and resistant cells were grown in microfluidic devices for 48 h at room temperature (21 to 24°C) under constant medium flow (tryptone broth at 0.1 μl/min). Biofilms were imaged immediately prior to phage treatment to establish exact starting ratios of wild-type cells (phage susceptible) and *trxA* deletion mutants (phage resistant). Subsequently, inlet medium tubing was removed from the PDMS microfluidic device, and new tubing containing phage diluted in tryptone broth (2 × 10^7^ PFU/ml at 0.1 μl/min) was inserted. Phage treatment continued for 1 h, after which original tubing was reinserted to resume flow of fresh tryptone broth without phages. Biofilms were imaged approximately 6, 12, 24, and 48 h after the conclusion of the phage treatment until a population dynamic steady state was reached.

**(vi) Calculation of *de novo* resistant mutants.** Biofilms of wild-type (i.e., susceptible) E. coli AR3110 were grown in microfluidic devices in identical conditions to primary experiments. After 48 h, outlet and inlet tubing were replaced with clean, unused tubing. The open end of the new outlet line was placed in a collection tube, while the open end of the inlet tubing was affixed to a 5-ml syringe containing tryptone broth. The 5-ml syringe was manually depressed with high pressure in order to shear the biofilm from the device. Visual inspection ensured clearance of the chamber. The collection effluent was then pelleted by centrifugation and resuspended in a known volume (200 μl). The sample was then serially diluted by pipetting 100 μl of sample into 900 μl of lysogeny broth. Eight serial dilutions were performed in this manner. From each dilution, 50 μl was aliquoted into two tubes. The first set of tubes received 100 μl of 1 × 10^12^ PFU/ml T7 phage, while the other set received 100 μl of blank media. The entire mixture was vortexed and plated on LB agar plates before incubating at 37°C overnight. Total CFU/chamber were calculated from the medium blank dilution series, while phage-resistant mutants were observed and counted in the set which received phage.

**(vii) Imaging and quantification procedures.** Biofilms were imaged using a Zeiss LSM 880 confocal microscope with a C-Apochromat 10×/0.45 water immersion objective or a 40×/1.2 water immersion objective. A 594-nm laser was used to excite mKate2, and a 543-nm laser line was used to excite mKOκ. A 640-nm laser was used to excite Alexa Fluor 633. Whole-chamber Z-stacks were acquired by utilizing 1 × 10 vertical tile scans (total rectangular area ∼500 × 5,000 μm). Quantification of biomass was performed using customized scripts in MATLAB (MathWorks, Natick, MA) as previously described by Drescher et al. (81), Nadell et al. (82), and Hartmann et al. (46). Biomass was used as a proxy for cell count, as the two strains have the same average cell size.
