# Supplementary figures and images for: Biofilm Structure Promotes Coexistence of Phage-Resistant and Phage-Susceptible Bacteria
Source: mSystems. 2020 Jun 23;5(3):e00877-19. doi: 10.1128/mSystems.00877-19 (PMC7311319; doi:10.1128/mSystems.00877-19)

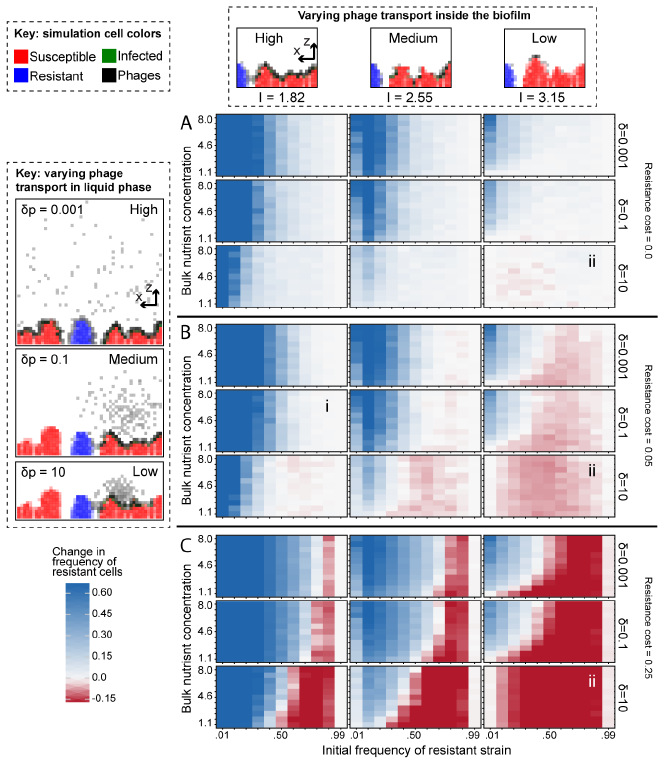

Supplement: FIG S1 [file mSystems.00877-19-sf001.tif]

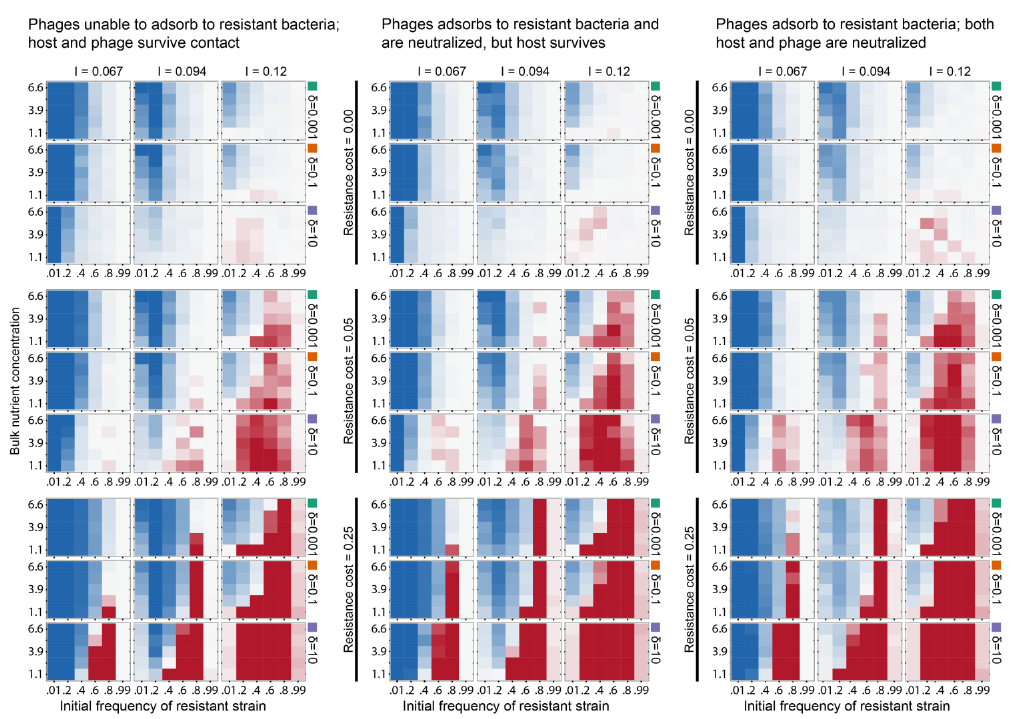

Supplement: FIG S2 [file mSystems.00877-19-sf002.tif]

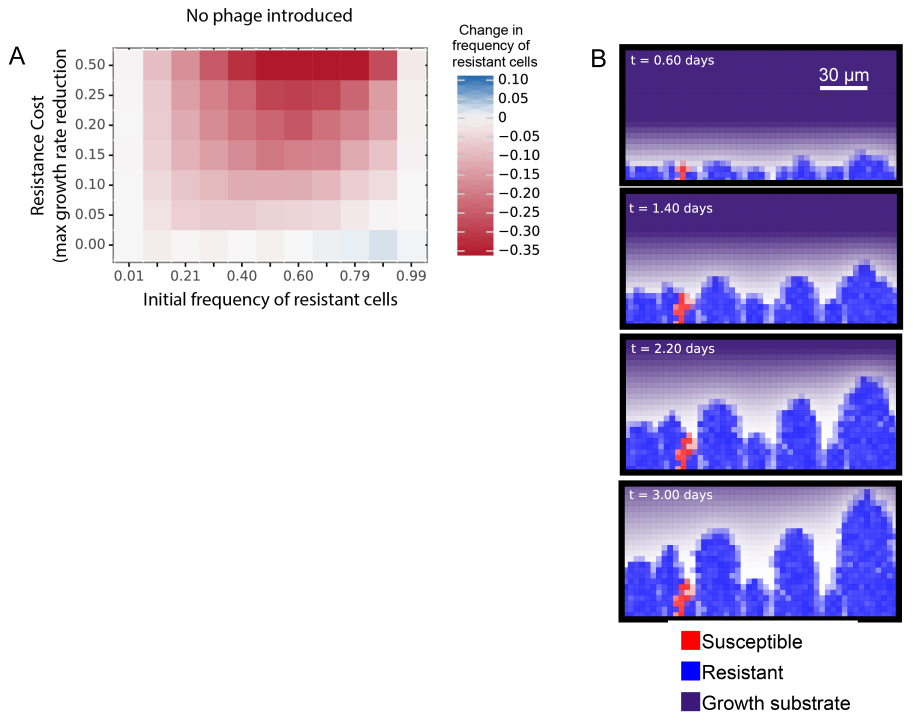

Supplement: FIG S3 [file mSystems.00877-19-sf003.tif]

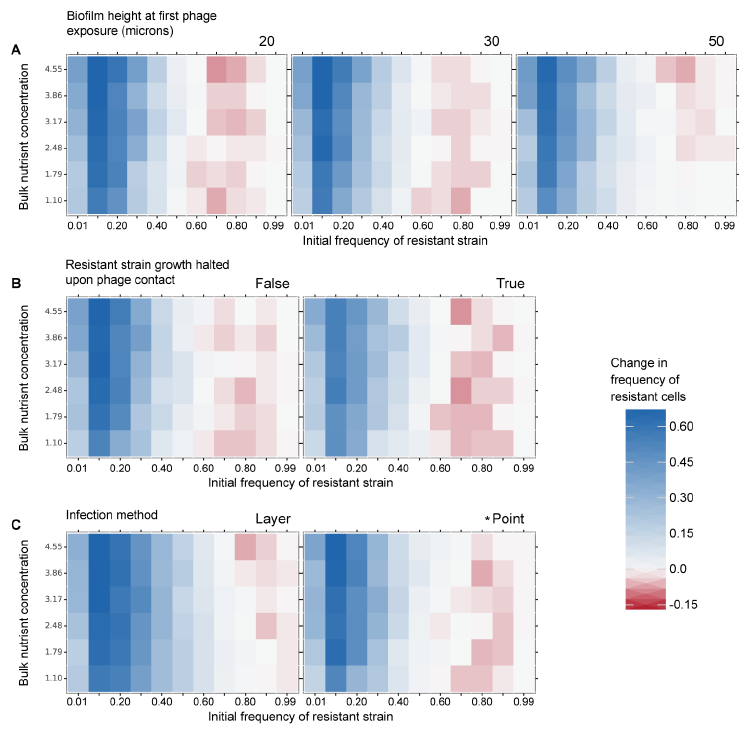

Supplement: FIG S4 [file mSystems.00877-19-sf004.tif]

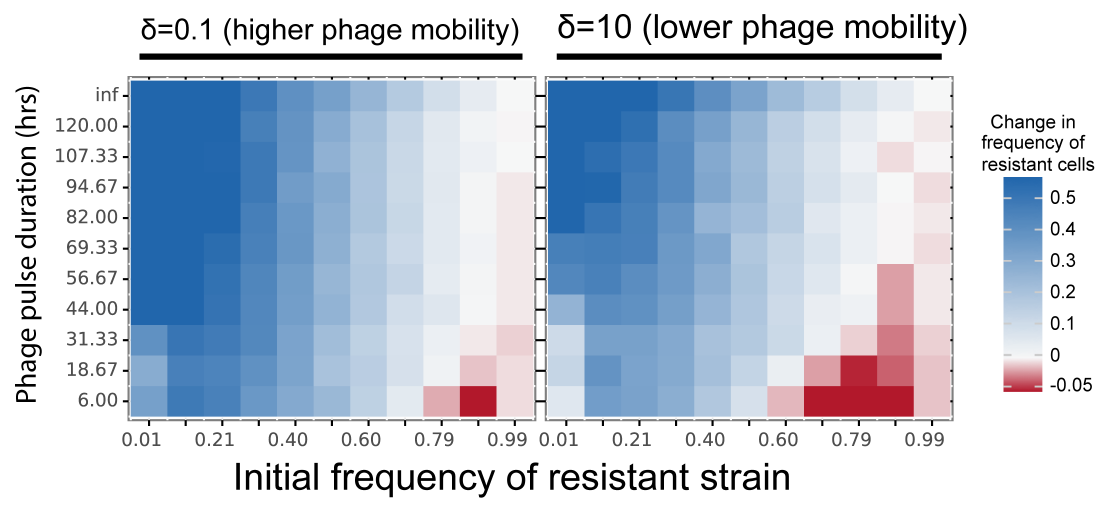

Supplement: FIG S5 [file mSystems.00877-19-sf005.tif]

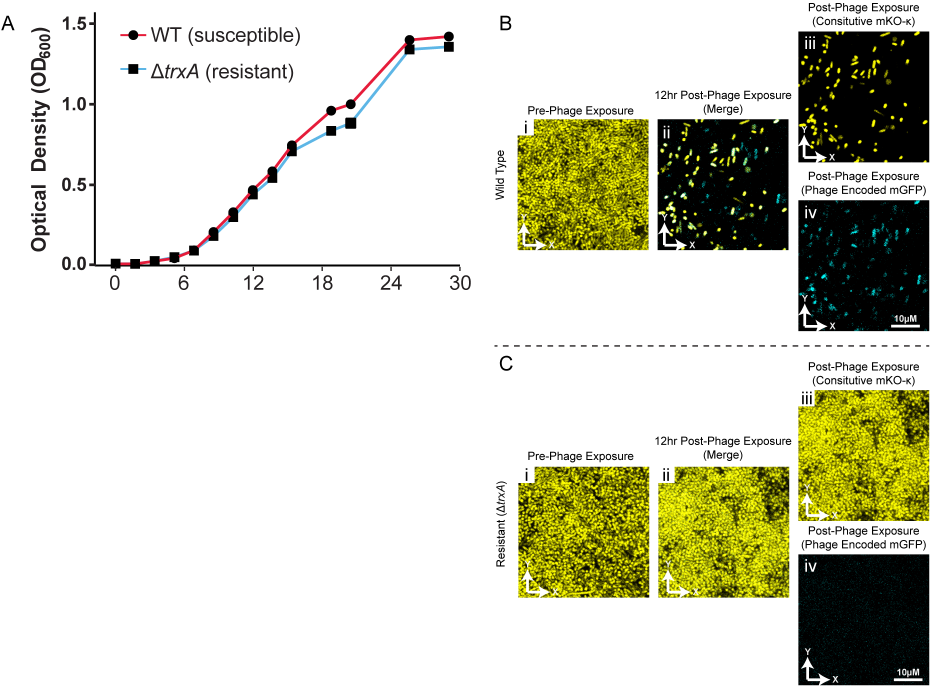

Supplement: FIG S6 [file mSystems.00877-19-sf006.tif]

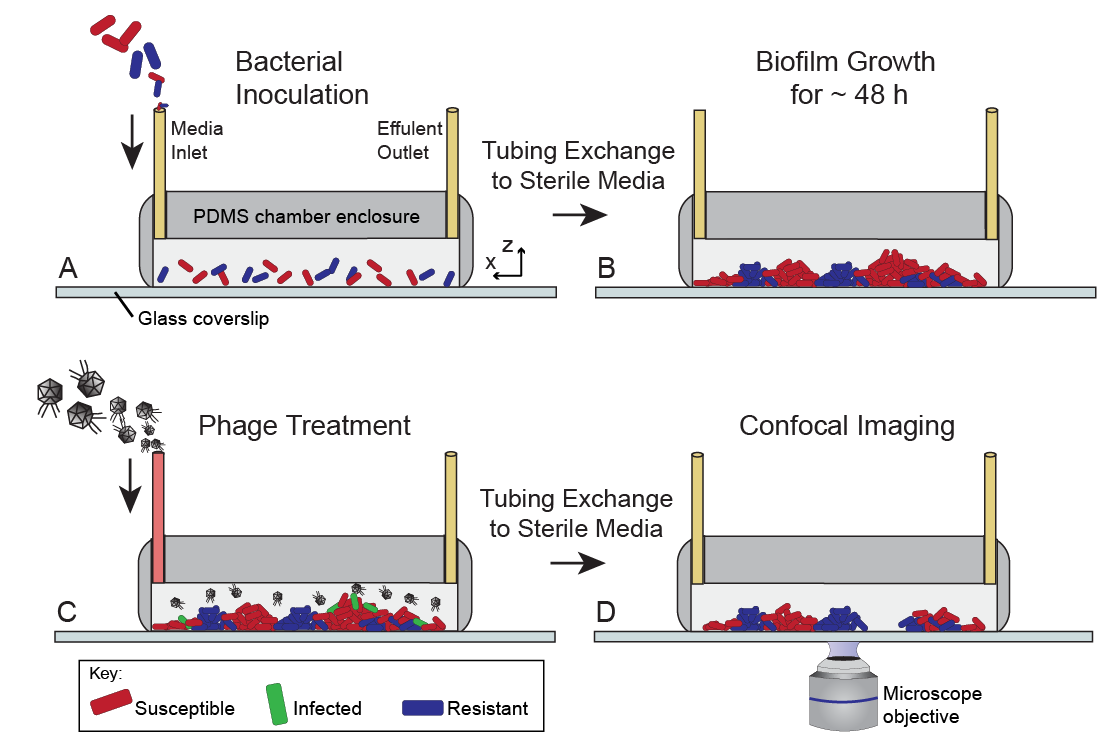

Supplement: FIG S7 [file mSystems.00877-19-sf007.tif]

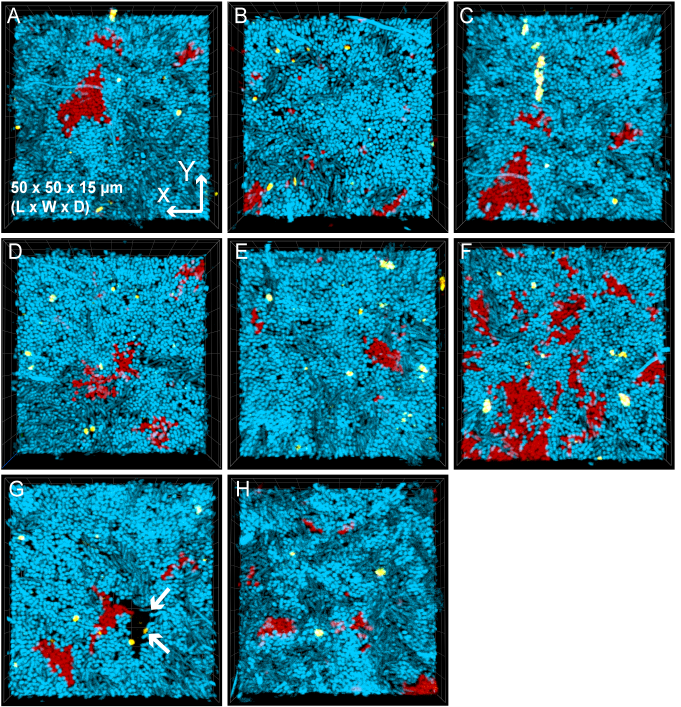

Supplement: FIG S8 [file mSystems.00877-19-sf008.tif]
